# Supplementary material for: Identification of trypanosomatids and blood feeding preferences of phlebotomine sand fly species common in Sicily, Southern Italy
Source: PLoS One. 2020 Mar 10;15(3):e0229536. doi: 10.1371/journal.pone.0229536 (PMC7064173; doi:10.1371/journal.pone.0229536)
Supplement: S1 Table — aTrypanossomatidae kinetoplast DNA. bHost mitochondrial DNA. cPCR product was previously diluted 1:50 in nuclease-free water. Abbreviations: cytB, cytochrome b; ITS-rDNA, ribosomal internal transcribed spacer DNA; SSU-rRNA, small subunit ribosomal DNA; bp, base pars. (DOCX) [file pone.0229536.s001.docx]

**S1 Table**

| **Target gene** | **Primer sequence (5’-3’)** | **Amplicon size** | **Reaction setup** | **Thermocycling conditions** | | **Reference** |
| --- | --- | --- | --- | --- | --- | --- |
| *cytB*^a^ | 1^st^ PCR | 919 bp | 25 µl reaction: 5 µl of DNA; 0.8 µM of each primer; 12.5 µl of NZYTaq 2 x Green Master Mix | 95 ºC - 3 min; 45 cycles [94 ºC - 1 min; 48 ºC - 1 min; 72 ºC - 1 min]; 72 ºC - 5 min | | (Pereira et al., 2019) |
|  | Fw: AGCGGAGAGRARAGAAAAGG |  |  |  |  |  |
|  | Rev: CTACAATAAACAAATCATAATATRCAATT |  |  |  |  |  |
|  | 2^nd^ PCR | 568 bp | 25 µl reaction: 5 µl of 1^st^ PCR product^b^; 0.8 µM of each primer; 12.5 µl of NZYTaq 2 x Green Master Mix | 95 ºC - 3 min; 45 cycles [94 ºC - 1 min; 48 ºC - 1 min; 72 ºC - 1 min]; 72 ºC - 5 min | |  |
|  | Fw: GGTGTAGGTTTTAGTYTAGG |  |  |  |  |  |
|  | Rev: GYTCRCAATAAAATGCAAATC |  |  |  |  |  |
|  |  |  |  |  | |  |
| *cytB*^b^ | Fw: CCATCCAACATYTCADCATGATGAAA | 360 bp | 25 µl reaction: 5 µl of DNA; 0.6 µM of each primer; 12.5 µl of NZYTaq 2 x Green Master Mix | 94 ºC - 5 min; 40 cycles [94 ºC – 1 min; 55 ºC – 1 min; 72 ºC – 1 min]; 72 ºC - 7 min | | (Maia et al., 2015) |
|  | Rev: GCHCCTCAGAATGATATTTGKCCTCA |  |  |  |  |  |
|  |  |  |  |  | |  |
| *ITS*-rDNA | Fw: CTGGATCATTTTCCGATG | 311 bp | 25 µl reaction: 2.5 µl of DNA; 0.4 µM of each primer; 12.5 µl of NZYTaq 2 x Green Master Mix | 95 ºC - 2 min; 32 cycles [95 ºC - 20 sec; 53 ºC - 30 sec; 72 ºC – 1 min]; 72 ºC - 6 min | | (El Tai et al., 2000) |
|  | Rev: TGATACCACTTATCGCACTT |  |  |  |  |  |
|  |  |  |  |  |  |  |
| *SSU*-rDNA | Fw: GAAACAAGAAACACGGGAG | 930 bp | 25 µl reaction: 5 µl of DNA; 0.4 µM of each primer; 12.5 µl of NZYTaq 2 x Green Master Mix | 95 ºC - 1 min; 40 cycles [95 ºC – 1 min; 55 ºC – 1 min; 72 ºC – 1 min]; 72 ºC - 10 min | | (Kato et al., 2011) |
|  | Rev: CTACTGGGCAGCTTGGA |  |  |  |  |  |

^a^Trypanossomatidae kinetoplast DNA

^b^Host mitochondrial DNA

^c^PCR product was previously diluted 1:50 in nuclease-free water

Abbreviations: *cytB,* cytochrome *b*; *ITS*-rDNA, ribosomal internal transcribed spacer DNA; *SSU*-rRNA, small subunit ribosomal DNA; bp, base pars
