# Supplementary material for: Identification of trypanosomatids and blood feeding preferences of phlebotomine sand fly species common in Sicily, Southern Italy
Source: PLoS One. 2020 Mar 10;15(3):e0229536. doi: 10.1371/journal.pone.0229536 (PMC7064173; doi:10.1371/journal.pone.0229536)
Supplement: S2 Table — A: LC469805; LC469817; LC469821; LC469832; LC469834; LC469836; LC469839; LC469842-44; LC469846-48; LC469850-51; LC469853-56; LC469863; LC469865; LC469871; LC469875-76; LC469878; LC469882; LC469887. B: LC469829; LC469837; LC469840-41; LC469852; LC469857; LC469859-62; LC469867; LC469872; LC469874; LC469880-81; LC469891-93; LC469896; LC469898; LC469900-03. C: LC469807-10; LC469813-16; LC469818-20; LC469822-26. D: LC469806; LC469811; LC469827-28; LC469830; LC469833; LC469835; LC469869; LC469877; LC469884, LC469890; LC469897; LC469908. E: LC469838; LC469845; LC469849; LC469858; LC469868; LC469870; LC469873; LC469883; LC469899. F: LC469885-86; LC469888-89; LC469905; LC469907; LC469910-12. G: LC469812; LC469906; LC469909. H: LC469831; LC469894; LC469904. I: LC469866. L: LC469879. M: LC469895. (DOCX) [file pone.0229536.s002.docx]

**S2** **Table.** **CytB sequences analysis for each sample, percentages of BLAST identity and sequences accession numbers.**

| **Blood meal source**  (scientific name) | **Sand fly species (n=176)** | | |  | | **Biotype** | |  | |  | | BLAST analysis | |  | |  |
| --- | --- | --- | --- | --- | --- | --- | --- | --- | --- | --- | --- | --- | --- | --- | --- | --- |
|  | *Phlebotomus perniciosus* | *Sergentomyia minuta* | *Phlebotomus sergenti* | |  | | Periurban | | Rural | |  | | Identity (%) | | Accession number | |
| Rabbit  (*Oryctolagus cuniculus*) | 27 | 1 |  | |  | |  | | 28 | |  | | 99-100 | | A | |
| Human  (*Homo sapiens*) | 8 | 16 |  | |  | | 11 | | 13 | |  | | 99-100 | | B | |
| Goat  (*Capra hircus*) | 16 |  |  | |  | | 1 | | 15 | |  | | 99-100 | | C | |
| Horse  (*Equus caballus*) | 11 | 2 |  | |  | | 4 | | 9 | |  | | 99-100 | | D | |
| Pig  (*Sus scrofa*) | 7 | 2 |  | |  | | 2 | | 7 | |  | | 99-100 | | E | |
| Dog  (*Canis familiaris*) | 8 | 1 |  | |  | | 1 | | 8 | |  | | 99-100 | | F | |
| Chicken  (*Gallus gallus*) | 3 |  |  | |  | |  | | 3 | |  | | 99-100 | | G | |
| Cow  (*Bos taurus*) | 1 | 2 |  | |  | | 2 | | 1 | |  | | 99-100 | | H | |
| Cat  (*Felis catus*) | 1 |  |  | |  | |  | | 1 | |  | | 99-100 | | I | |
| Donkey  (*Equus asinus*) |  | 1 |  | |  | |  | | 1 | |  | | 99-100 | | L | |
| Rat  (*Rattus norvegicus*) |  |  | 1 | |  | | 1 | |  | |  | | 99-100 | | M | |
| **Total (identified blood meals)** | 82 | 25 | 1 | |  | | 22 | | 86 | |  | |  | |  | |
| **Total (Non-identified blood meals)** | 56 | 12 |  | |  | |  | |  | |  | |  | |  | |

**A**: LC469805; LC469817; LC469821; LC469832; LC469834; LC469836; LC469839; LC469842-44; LC469846-48; LC469850-51; LC469853-56; LC469863; LC469865; LC469871; LC469875-76; LC469878; LC469882; LC469887. **B**: LC469829; LC469837; LC469840-41; LC469852; LC469857; LC469859-62; LC469867; LC469872; LC469874; LC469880-81; LC469891-93; LC469896; LC469898; LC469900-03. **C**: LC469807-10; LC469813-16; LC469818-20; LC469822-26. **D**: LC469806; LC469811; LC469827-28; LC469830; LC469833; LC469835; LC469869; LC469877; LC469884, LC469890; LC469897; LC469908. **E**: LC469838; LC469845; LC469849; LC469858; LC469868; LC469870; LC469873; LC469883; LC469899. **F**: LC469885-86; LC469888-89; LC469905; LC469907; LC469910-12. **G**: LC469812; LC469906; LC469909. **H**: LC469831; LC469894; LC469904. **I**: LC469866. **L**: LC469879. M: LC469895
