# Supplementary material for: Identification of trypanosomatids and blood feeding preferences of phlebotomine sand fly species common in Sicily, Southern Italy
Source: PLoS One. 2020 Mar 10;15(3):e0229536. doi: 10.1371/journal.pone.0229536 (PMC7064173; doi:10.1371/journal.pone.0229536)
Supplement: S3 Table — (DOCX) [file pone.0229536.s003.docx]

| ***Phlebotomus perniciosus* blood meal sources** | | | | | | | | | |
| --- | --- | --- | --- | --- | --- | --- | --- | --- | --- |
|  | *Capra hircus* | *Canis familiaris* | *Equus caballus* | *Sus scrofa* | | *Homo sapiens* | *Gallus gallus* | *Bos taurus* | *Felis catus* |
| *Oryctolagus cuniculus* | *χ2* = 4.8,  *P* = 0.01 | *χ2* = 13.6,  *P* < 0.001 | *χ2* = 15.3,  *P* <0.001 | *χ2* = 17.3,  *P* <0.001 | | *χ2* = 19.4,  *P* <0.001 | *χ2* = 15.3,  *P* <0.001 | *χ2* = 24.2,  *P* <0.001 | *χ2* = 24.2,  *P* <0.001 |
| *Capra hircus* |  | *χ2* = 2.5,  *P* = 0.06 | *χ2* = 3.4,  *P* = 0.03 | *χ2* = 4.5,  *P* = 0.01 | | *χ2* = 5.8,  *P* = 0.008 | *χ2* = 9.1,  *P* = 0.001 | *χ2* = 24.2,  *P* <0.001 | *χ2* = 13.8,  *P* <0.001 |
| *Canis familiaris* | *χ2* = 2.5,  *P* = 0.06 |  | *χ2* = 0.07,  *P* = 0.39 | *χ2* = 0.32,  *P* = 0.29 | | *χ2* = 0.8,  *P* = 0.19 | *χ2* = 2.5,  *P* = 0.06 | χ2 = 5.8,  *P* = 0.008 | *χ2* = 5.8,  *P* = 0.008 |
| *Equus caballus* | *χ2* = 3.4,  *P* = 0.02 | *χ2* = 0.07,  *P* = 0.39 |  | χ2 = 0.08,  *P* = 0.38 | | *χ2* = 0.36,  *P* = 0.27 | *χ2* = 1.7,  *P* = 0.09 | *χ2* = 4.7,  *P* = 0.01 | *χ2* = 4.8,  *P* = 0.01 |
| *Sus scrofa* | *χ2* = 4.5,  *P* = 0.01 | *χ2* = 0.32,  *P* = 0.29 | *χ2* = 0.8,  *P* = 0.40 |  | | *χ2* = 0.09,  *P* = 0.37 | *χ2* = 1.1,  *P* = 0.15 | *χ2* = 3.7,  *P* = 0.02 | *χ2* = 3.8,  *P* = 0.03 |
| *Homo sapiens* | *χ2* = 5.8,  *P* = 0.008 | *χ2* = 0.8,  *P* = 0.19 | χ2 = 0.36,  *P* = 0.27 | *χ2* = 0.09,  *P* = 0.37 | |  | χ2 = 2.7,  *P* = 0.04 | *χ2* = 2.7,  *P* = 0.04 | *χ2* = 2.8,  *P* = 0.04 |
| *Gallus gallus* | *χ2* = 9.1,  *P* = 0.001 | *χ2* = 2.5,  *P* = 0.06 | χ2 = 1.7,  *P* = 0.09 | *χ2* = 1.1,  *P* = 0.15 | | *χ2* = 0.5,  *P* = 0.2 |  | *χ2* = 1.3,  *P* = 0.15 | *χ2* = 1.0,  *P* = 0.15 |
| *Bos taurus* | *χ2* = 13.8,  *P* <0.001 | *χ2* = 5.8,  *P* = 0.008 | *χ2* = 4.7,  *P* = 0.01 | *χ2* = 3.7,  *P* = 0.02 | | *χ2* = 2.7,  *P* = 0.04 | *χ2* = 1.3,  *P* = 0.15 |  | *χ2* = 0.9,  *P* = 0.50 |
| ***Sergentomyia minuta* blood meal sources** | | | | | | | | | |
|  | *Equus asinus* | *Equus caballus* | *Homo sapiens* | | *Sus scrofa* |  |  |  |  |
| *Oryctolagus cuniculus* | *χ2* = 0.9,  *P* = 0.5 | *χ2* = 0.4,  *P* = 0.3 | *χ2* = 8.3,  *P* = 0.02 | | *χ2* = 0.9,  *P* = 0.5 |  |  |  |  |
| *Equus asinus* |  | *χ2* = 0.4,  *P* = 0.3 | *χ2* = 8.3,  *P* = 0.02 | | *χ2* = 0.9,  *P* = 0.5 |  |  |  |  |
| *Equus caballus* | *χ2* = 0.4,  *P* = 0.3 |  | *χ2* = 5.9,  *P* = 0.007 | | *χ2* = 0.4,  *P* = 0.3 |  |  |  |  |
| *Homo sapiens* | *χ2* = 8.3,  *P* = 0.02 | *χ2* = 5.9,  *P* = 0.007 |  | | *χ2* = 8.3,  *P* = 0.02 |  |  |  |  |

**S3 Table. Differences in host preference within the most abundant sand fly species caught in site E.**
